# Exploring Environmental Influences on Mono(2‐Ethylhexyl) Phthalate (MEHP) Exposure in Common Bottlenose Dolphins (*Tursiops truncatus*) in Sarasota Bay, FL, USA

**DOI:** 10.1029/2026GH002199

**Published:** 2026-11-06

**Authors:** Kylie B. Warden, Miranda K. Dziobak, Krystan A. Wilkinson, Randall S. Wells, Paul A. Sandifer, Vijay M. Vulava, Leslie B. Hart

**Affiliations:** ^1^ Environmental and Sustainability Studies Graduate Program College of Charleston Charleston SC USA; ^2^ Department of Public Health Sciences and Administration School of Health Sciences College of Charleston Charleston SC USA; ^3^ Department of Environmental Health Sciences Arnold School of Public Health University of South Carolina Columbia SC USA; ^4^ Sarasota Dolphin Research Program Sarasota FL USA; ^5^ Center for Coastal Environmental and Human Health College of Charleston Charleston SC USA; ^6^ Department of Geology and Environmental Geosciences School of Natural and Environmental Sciences College of Charleston Charleston SC USA

**Keywords:** phthalates, organic pollutants, environmental contamination, bottlenose dolphins, marine mammals

## Abstract

Phthalates, endocrine‐disrupting, persistent organic pollutants widely used in plastic manufacturing, can readily enter aquatic environments and contaminate estuaries. As apex predators, resident bottlenose dolphins (*Tursiops truncatus*) provide critical insights into ecosystem health. Prior work documented phthalate exposure in the long‐term resident bottlenose dolphin community of Sarasota Bay, Florida, with evidence of temporal and spatial variability, particularly for mono(2‐ethylhexyl) phthalate (MEHP), a metabolite of the commonly used plasticizer di(2‐ethylhexyl) phthalate (DEHP). We predicted that environmental factors, such as large‐scale storm events, rainfall, and harmful algal blooms (HABs), may influence this variability. Urine samples collected from 138 free‐ranging dolphins during 1993–2024 were screened for phthalate metabolites, reliable biomarkers of exposure. MEHP was the most common metabolite, detected in 78.3% of samples (Regression on Order Statistics mean = 6.19 ng mL^−1^, s.d. = 11.29 ng mL^−1^, range = 0.04–76.6 ng mL^−1^), and concentrations did not vary significantly by sex or age (*p* > 0.05). MEHP concentrations were positively correlated with rainfall occurring 3 days prior to sampling (*p* = 0.01), and concentrations were significantly lower among dolphins sampled within 6 months of a major storm event (*p* = 0.007). No significant associations between MEHP and HABs were discerned (*p* > 0.05). Such findings suggest increased phthalate‐contaminated runoff during short‐term rainfall events, as well as the dilution or offshore transport of phthalates via storm‐related processes. Despite documented toxicity of MEHP to humans and other marine species, health impacts on Sarasota Bay dolphins remain poorly understood. As sentinels of estuarine health, long‐term exposure may signal chronic ecotoxicological risks, and observed environmental influences highlight the need for continued monitoring, research, and mitigation.

## Plain Language Summary

Past work found differences in plastic‐related phthalate pollution exposure over time and across geographic areas among bottlenose dolphins (*Tursiops truncatus*) residing in Sarasota Bay, Florida. To help explain these differences, this study aimed to understand the impacts of three common natural meteorological and environmental occurrences (large‐scale storms, short‐term rainfall, and harmful algal blooms [HABs]) on phthalate exposure in Sarasota Bay dolphins over 30 years. Data from phthalate metabolites measured in archived urine samples from 138 dolphins were used for analysis. Mono(2‐ethylhexyl) phthalate (MEHP) was the most frequently detected metabolite across urine samples, and levels did not differ by dolphin sex or age. MEHP levels increased when rainfall occurred within 3 days of urine sampling and decreased in dolphin urine sampled within 6 months of a major storm. These findings may reflect increased pollutant runoff from short‐term rainfall, as well as the dilution of estuarine waters or movement of phthalates into more open waters driven by large‐scale storms. MEHP levels were not associated with HAB events. Overall, these findings suggest that phthalate exposure is widespread in long‐term resident dolphins, and rainfall and major storms may influence exposure, highlighting the need for further research on sources of estuarine phthalate exposure and contamination.

## Key Points

- Mono(2‐ethylhexyl) phthalate exposure is persistent among Sarasota Bay bottlenose dolphins over time, regardless of age or sex
- Short‐term rainfall may facilitate acute phthalate exposure events in Sarasota Bay bottlenose dolphins, while large‐scale storm events may reduce phthalate contamination within this area
- Long‐term environmental trends in dolphin phthalate exposure could reflect estuarine contamination in Sarasota Bay

## 1 Introduction

### 1.1 Phthalate Background

Plastic pollution is a global concern due to its environmental ubiquity, resistance to degradation, and adverse health implications. For example, in 2020, global plastic production reached 464 million tonnes, with production expected to double by 2050 (Dokl et al., 2024). Global plasticizer production exceeded 8 million tonnes in 2020 and is predicted to reach nearly 13 million tonnes by 2030 (ChemAnalyst, 2022). Among these plasticizers, phthalates are the most common, accounting for around 85% of the global plasticizer market (Godwin, 2017).

Phthalates are chemical additives extensively used in domestic and industrial manufacturing. High‐molecular‐weight phthalates, including di(2‐ethylhexyl) phthalate (DEHP), are often added to plastics (Agency for Toxic Substances and Disease Registry [ATSDR], 2022), while low‐molecular‐weight phthalates, like diethyl phthalate (DEP), are frequently used as solvents in personal care products (ATSDR, 1995; Koniecki et al., 2011). While regulations on DEHP have led to industry replacements, this phthalate remains the dominant plasticizer, comprising approximately 40% of all plasticizer production (Qu et al., 2022).

These plasticizers are not chemically bound to the product to which they are added, so they can readily leach into the surrounding environment (Heudorf et al., 2007; Paluselli et al., 2019). Higher‐molecular‐weight phthalates, like DEHP, are highly lipophilic and hydrophobic and are, therefore, more likely to partition to sediments and accumulate in fatty tissues of animals (Paluselli & Kim, 2020; Staples et al., 1997; L. Y. Wang et al., 2021). Phthalates are also rapidly metabolized by microbes in the environment (Staples et al., 1997; Sun et al., 2019), where metabolites can also contaminate upon exposure and excreted in urine, providing a reliable biomatrix for evaluating exposure in humans and wildlife.

As such, phthalates are ubiquitous in aquatic and marine environments (Li et al., 2024; Z. M. Zhang et al., 2020), contaminating these systems through wastewater discharge (Kotowska et al., 2020; Tran et al., 2015), agricultural applications (Hui et al., 2021; Niu et al., 2014), urban runoff (Hajiouni et al., 2022; Wicke et al., 2021), landfill leachates (Gholaminejad et al., 2024; Mohammadi et al., 2022), plastic debris degradation (Henkel et al., 2023; Paluselli et al., 2019), and atmospheric deposition (Kang et al., 2017; Mi et al., 2023). Proximity to urbanization and anthropogenic activity can also influence marine phthalate inputs and distribution (Cao et al., 2022; L. Y. Wang et al., 2021; Weizhen et al., 2020).

### 1.2 Phthalate Exposure in Common Bottlenose Dolphins

Common bottlenose dolphins (*Tursiops truncatus*) residing in estuaries are sentinels of marine ecosystem health (Kucklick et al., 2011; Wells et al., 2004) and have been used as indicators of environmental contamination from legacy persistent organic pollutants (Kucklick et al., 2022), oil spills (Schwacke et al., 2014), and harmful algal blooms (HABs; Fire et al., 2008). The Sarasota Dolphin Research Program (SDRP) has studied the biology, ecology, physiology, and behavior of the long‐term resident common bottlenose dolphins in Sarasota Bay, FL, USA, since 1970 (Wells, 2009, 2014). Recent studies of urine samples collected from dolphins inhabiting Sarasota Bay have also identified prevalent exposure to multiple phthalate compounds (Dziobak et al., 2021, 2025; Hart et al., 2018), with some metabolite concentrations exceeding those reported for human reference populations (Hart et al., 2020).

Potential health impacts of phthalates on humans are well‐documented (Savoca et al., 2025). For instance, human and laboratory studies on rodents suggest that phthalate exposure can alter gene expression, resulting in oxidative stress, metabolic disorders, immunosuppression, endocrine disruption, and apoptosis or cell death (Y. Zhang et al., 2021). In sensitive aquatic model organisms, neurological (Y. Liu et al., 2023), endocrine (Mankidy et al., 2013), reproductive (Ye et al., 2014), immune (S. Wang et al., 2020), genetic (Molino et al., 2019), and developmental toxicity (Seyoum & Pradhan, 2019) have been observed across life stages in freshwater and saltwater laboratory studies. In marine mammals, recent studies have documented correlations between aldosterone concentrations and phthalate detection in aquarium‐housed killer whales (Lemos et al., 2024) and between free thyroxine and MEHP concentrations in the wild Sarasota Bay dolphin community (Dziobak, Wells, et al., 2022). However, current knowledge of long‐term phthalate‐related health risks in marine mammals remains limited despite high detection of these contaminants.

Among Sarasota Bay dolphins sampled during 2010–2024, approximately 75% (95% Confidence Interval [CI] = 61.41–83.06%; *n* = 71) had detectable urinary phthalate metabolite concentrations (i.e., concentrations > limits of detection [LOD]), with mono(2‐ethylhexyl) phthalate (MEHP) and monoethyl phthalate (MEP) being the most prevalent, demonstrating high environmental exposure to parent compounds DEHP and DEP (Dziobak et al., 2025). No significant differences were reported between sex or age class, indicating that all resident dolphins inhabiting Sarasota Bay are likely at risk of exposure (Dziobak et al., 2021, 2025; Hart et al., 2018). Despite no discernible demographic predisposition to phthalate exposure, spatiotemporal comparisons revealed unexplained variability in MEHP exposure among these dolphins (Dziobak, Balmer, et al., 2022), suggesting external factors (e.g., climate, land use) could influence exposure.

### 1.3 Environmental Influences of Estuarine Phthalate Contamination

Environmental influences on aquatic and marine phthalate contamination have been documented (C. F. Chen et al., 2022; Heo et al., 2020; N. Liu et al., 2016; Sun et al., 2019), but the impact of environmental factors on phthalate exposure in aquatic and marine species remains poorly understood. Sarasota Bay is a highly urbanized estuary (Leverone et al., 2017) susceptible to natural disasters, including tropical storms and hurricanes (Carlson et al., 2010; Tomasko et al., 2020), intense rainfall and droughts (Irizarry‐Ortiz et al., 2011; SBEP, 2017), and HABs (Heil & Muni‐Morgan, 2021; Weisberg et al., 2019). From 2020 through May 2024, Sarasota Bay has experienced passing effects from five hurricanes and one unusually heavy rainfall event (National Weather Service [NWS], n.d.), and a minimum of four HAB events reaching fish kill levels (Florida Fish and Wildlife Conservation Commission‐Fish and Wildlife Research Institute [FFWCC‐FWRI], 2018).

Large‐scale storm events can increase turbulent conditions, facilitating phthalate leaching from plastics (Henkel et al., 2023; Suhrhoff & Scholz‐Böttcher, 2016) and resuspending pollutants from sediments (Billings et al., 2024). Elsewhere, reduced phthalate concentrations in estuarine waters have been associated with the rainy season, indicating contaminant dilution (Ai et al., 2021; C. F. Chen et al., 2022; Heo et al., 2020). For example, phthalate concentrations in seawater declined more than 80% along the southeast coast of South Korea following nearly one inch of rainfall in a single day compared to pre‐rainfall concentrations (Heo et al., 2020). However, positive correlations between precipitation and phthalate contamination have also been observed during the rainy season and linked to atmospheric deposition (Gao & Wen, 2016), land use (Cao et al., 2022; Wicke et al., 2021), and pollutant runoff (Müller et al., 2019; Weizhen et al., 2020). The Sarasota Bay area also has a history of outdated wastewater infrastructure and stormwater management (SBEP, 2014) and is one of the fastest‐growing areas in the Southeast US (Thompson & Fernandez, 2026). Storm surge and heavy rainfall can overwhelm aging wastewater and septic systems, polluting the Bay with excess nutrients (D. Chen et al., 2019; Mendoza, 2024; Salman, 2025; SBEP, 2022b, 2026) and potentially compounding estuarine phthalate contamination (Kotowska et al., 2020; Soleimani et al., 2025). While stormwater management has improved over the years, increasing development and population growth continue to exacerbate pollutant runoff in the Bay (SBEP, 2022a), which may contain phthalates (Cao et al., 2022; L. Y. Wang et al., 2021; Weizhen et al., 2020).

Excess nutrient inputs from these sources can also trigger HABs (Salman, 2025) in the Gulf of Mexico. HABs, when defined as cell counts >100,000 cells L^−1^, occur annually and generally last, on average, over five months in the Sarasota Bay area (Tomasko et al., 2024). Phthalates in these systems can interact with algae, including *Karenia brevis*, a dinoflagellate responsible for some HABs in the Gulf of Mexico. For instance, phthalates can restrict the cell growth and photosynthesis of marine algae (Savoca et al., 2026), *K*. *brevis* included (N. Liu et al., 2016). However, *K. brevis* has also been shown to degrade low‐molecular‐weight phthalates and phthalic acid by up to 93.3% over 96 hr (Sun et al., 2019). While algae can degrade phthalates, they can also release secondary phthalate metabolites during biodegradation (Savoca et al., 2026), potentially increasing phthalate bioavailability in marine environments. HABs can also cause fish kills that shift prey availability for higher trophic organisms (Berens McCabe et al., 2021; Gannon et al., 2009; Wells et al., 2013), potentially influencing phthalate exposure through trophic dynamics. In Sarasota Bay, HABs significantly altered prey composition (Berens McCabe et al., 2021) and common bottlenose dolphin (*Tursiops truncatus*) foraging behavior toward more open bay waters (McHugh et al., 2011). Such prey shifts, combined with the complex interactions between phthalates and algal primary producers, could affect phthalate exposure in bottlenose dolphins and other higher trophic organisms. Overall, these studies suggest that heavy rainfall, tropical storms, tropical depressions, and hurricanes (hereafter referred to as storm events) and HABs may alter phthalate fate in marine ecosystems, particularly in Sarasota Bay where these events are frequent.

### 1.4 Objective

Sarasota Bay, FL, is a storm‐ and HAB‐prone, subtropical, and highly developed estuary. Its resident dolphin community has been the subject of long‐term research efforts by the SDRP (Wells, 2014), including studies on phthalate exposure (Dziobak et al., 2021, 2025; Hart et al., 2018). Information from these previous studies, as well as the availability of urine samples from dolphin health assessments over 30 years, provided the opportunity to explore environmental linkages with phthalate exposure. Therefore, the objective of this study was to determine whether three environmental factors (i.e., storm events, rainfall, HAB events) common to Sarasota Bay could influence phthalate exposure in the long‐term resident bottlenose dolphin community. We hypothesized that storm events and short‐term rainfall would be positively associated with MEHP exposure, as evidence indicates that these weather events exacerbate runoff and atmospheric deposition of phthalates into estuaries (Cao et al., 2022; Gao & Wen, 2016; Müller et al., 2019; Weizhen et al., 2020; Wicke et al., 2021). We also hypothesized that HAB events would influence MEHP exposure in Sarasota Bay bottlenose dolphins due to related changes in foraging (McHugh et al., 2011) and prey availability (Berens McCabe et al., 2021; Gannon et al., 2009; Wells et al., 2013), as well as the contrasting interactions between HABs and phthalates (N. Liu et al., 2016; Sun et al., 2019).

## 2 Materials and Methods

### 2.1 Study Site

The study area was the central west coast of Florida, including Sarasota Bay and adjacent estuarine waters within Manatee and Sarasota counties, extending approximately 40 km north to south and 1 km offshore of the barrier islands (Figure 1; Scott et al., 1990). Consisting of approximately 170 year‐round resident individuals, the Sarasota Bay bottlenose dolphin community inhabits shallow bays, seagrass meadows, and mangrove habitats, as well as Gulf coast waters off the surrounding barrier islands, and ranges from southern Tampa Bay to Venice Inlet (Figure 1). These resident dolphins interact socially throughout their lives (Wells, 2014; Wells & McHugh, 2025) and exhibit strong site fidelity to the Bay following environmental disturbances (Bassos‐Hull et al., 2013; McHugh et al., 2011). This, combined with their low emigration and immigration rates (Wells, 2014; Wells et al., 2025), makes them ideal candidates for long‐term, multigenerational studies. The SDRP's extensive history of bottlenose dolphin research (Wells et al., 2013) enables the assessment of contaminant exposure across life histories, demographics, and spatiotemporal scales (Wells et al., 2005).

**Figure 1:**
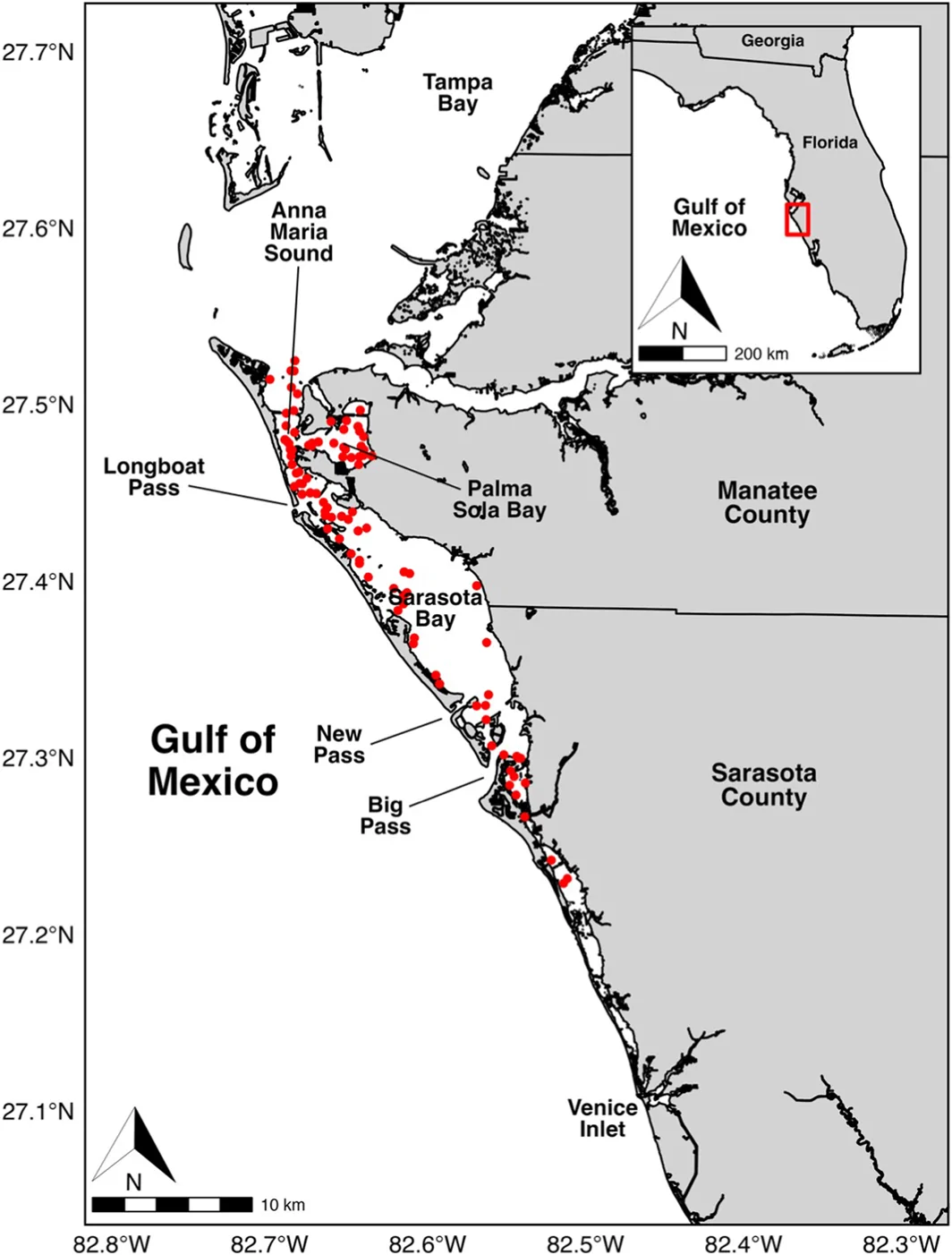
Study area near Sarasota, FL. The points represent the locations where urine samples used in this study were collected during routine catch‐and‐release health assessments.

### 2.2 Phthalate Metabolite Data Collection and Analysis

#### 2.2.1 Urine Sampling

This research utilized a long‐term data set (1993–2024; *n* = 138) of urine samples previously collected via catheter from 138 individual free‐ranging resident bottlenose dolphins during routine catch‐and‐release health assessments (Figure 1). All samples from this study were retrieved from the SDRP sample archives and the National Marine Mammal Tissues Bank housed at the National Institute of Standards and Technology (NIST) Biorepository (Dziobak et al., 2021). Details on urine and blank collection, transport, and storage have been reported by Hart et al. (2018). Matched field blank samples were collected beginning in 2016 following methods described by Dziobak et al. (2021) and Hart et al. (2018). Urine is the standard matrix used to monitor phthalate exposure in humans (National Center for Environmental Health [NCEH], 2021; National Health and Nutrition Examination Survey [NHANES], 2021). Urinary phthalate metabolites provide accurate and representative indications of phthalate exposure, given the rapid metabolism and excretion of monoesters, the consolidation of concentrations across different exposure pathways, the sensitive analytical detection of metabolites, and the minimized potential for contamination during sampling and analysis (Blount et al., 2000; Calafat & McKee, 2006). Urine samples were collected during health assessments conducted by the SDRP under Scientific Research Permit Numbers 655, 945, 522–2569, 522–1785, 15543, 20455, and 26622 issued by the National Oceanic and Atmospheric Administration's (NOAA) National Marine Fisheries Service. Sample collection was reviewed and approved annually by Mote Marine Laboratory's Institutional Animal Care and Use Committee. These health assessments have been previously described by Wells et al. (2004) and Barratclough et al. (2019). Because the time it takes for phthalates to be excreted from dolphins has not yet been studied, this study adhered to the protocols described in Dziobak, Balmer, et al. (2022) and used a single urine sample as a conservative estimate of metabolite concentrations over a 12‐month period. Accordingly, as dolphin health assessments are typically conducted in May or June each year, urinary phthalate metabolite concentrations were limited to the time of sampling.

#### 2.2.2 Phthalate Metabolite Screening

Urine samples were previously screened for eight phthalate metabolites by two separate analytical laboratories. These metabolites included MEHP, MEP, monomethyl phthalate (MMP), mono‐2‐ethyl‐5‐hydroxyhexyl phthalate (MEHHP), mono‐(2‐ethyl‐5‐oxohexyl) phthalate (MEOHP), monobenzyl phthalate (MBzP), monoisobutyl phthalate (MiBP), and monobutyl phthalate (MBP). Phthalate metabolites were isolated from urine collected during 1993–2009 (*n* = 67) and analyzed by a commercial laboratory (Creative Proteomics, Shirley, NY; later referred to as Lab 1) in 2024. These samples, which were new to this study, were analyzed using liquid‐liquid extraction methods and liquid chromatography‐tandem mass spectrometry (LC‐MRM/MS). Lab 1 processed and spiked 400 μL of urine and reagent blank samples and analyzed 20 μL aliquots by LC‐MRM/MS. Samples from 2010 to 2024 (*n* = 71), as reported by Dziobak et al. (2021; later referred to as Lab 2), were analyzed using solid‐phase extraction and quantified by high‐performance liquid chromatography combined with triple quadrupole mass spectrometry and electrospray ionization interface (HPLC‐ESI/MS). Briefly, 1 mL of urine samples was spiked, and reagent blanks, reagent spikes, matrix spikes, positive controls, and field blanks were processed with the urine samples (2021). Analyses of these samples and quality assurance/quality control have been previously described and reported (Dziobak et al., 2025). In both laboratories, urinary metabolite concentrations were quantified using calibration curves and corrected using reagent blanks (Dziobak et al., 2025). No samples in this study were analyzed by both labs. While urine samples were analyzed by two different laboratories with varying sensitivities and limits of detection (LOD; Tables S1 and S2 in Supporting Information S1), we conducted concentration‐based analysis rather than examining associations relative to detection status (i.e., detect vs. non‐detect) to account for variations in lab sensitivity.

### 2.3 Environmental Data Collection

To examine the linkages between environmental factors and phthalate exposure in Sarasota Bay resident bottlenose dolphins, urinary metabolite concentrations were evaluated in relation to average antecedent rainfall, storm events, and HAB events that have occurred in Sarasota and Manatee counties since 1992. Continuous daily rainfall data were collected from NOAA's National Center for Environmental Information (NCEI) Climate Monitoring Database for Sarasota County (Myakka River State Park, FL station; 27.24012°N, 82.31525°W [WGS, 84]), and 3‐day and 15‐day average antecedent rainfall were calculated prior to a health assessment event to assess short‐term hydrological trends, similar to Hitchcock (2020). Although located approximately 24 km inland between the cities of Sarasota and Venice (south of Sarasota) and does not drain into Sarasota Bay, this weather station was used for its consistent rainfall measurements covering the entire study period (1993–2024). This area also still impacts the Bay through rain‐induced influx.

While effects from large‐scale storm events were also considered, average antecedent rainfall was included to examine the potential influence of short‐term effects of rainfall intensity, as the magnitude of storm events (e.g., hurricane category, tropical storm) was not accounted for here. A storm event was defined as any tropical storm, tropical depression, or hurricane that was documented within Sarasota or Manatee County by ESRI's NOAA Storm Events Database Explorer, from which storm event data for 1996–2024 were obtained. Tropical storms, tropical depressions, and hurricanes during 1992–1995 were obtained from NOAA's Historical Hurricane Tracks and the National Weather Service. Using these data, binary variables (Yes/No) were generated to indicate whether each sample date occurred within six, nine, or 12 months of a storm event. These time intervals were defined to streamline analyses, cover the hurricane season in Sarasota Bay (June–October) relative to the timing of dolphin health assessments (typically May/June), and remain consistent with the previously described protocols for estimating phthalate metabolite concentrations over a 1‐year timeframe.

A HAB event was defined as a period where *K. brevis* cell counts exceeded fish kill levels (100,000 cells L^−1^; Landsberg & Steidinger, 1998; Quick & Henderson, 1974) for at least 15 days, preceded by a minimum of 60 days without consecutive fish kill levels. These methods aligned with the HAB events determined in Berens McCabe et al. (2021). HAB data were collected from the FFWCC's HAB Monitoring Database and NOAA NCEI Harmful Algal BloomS Observing System (HABSOS), and data were restricted to two miles offshore in Sarasota and Manatee counties based on known resident dolphin ranging patterns (Irvine et al., 1981; Scott et al., 1990). As HABs occur year‐round, these data were used to generate binary variables (Yes/No) denoting whether each sample date occurred within three, six, nine, or 12 months of a HAB event.

### 2.4 Statistical Analysis

All statistical analyses were conducted using R software (version 4.5.1; R Core Team, 2024), and all figures were generated in R. Given that MEHP has been the most detected phthalate metabolite among Sarasota Bay bottlenose dolphins in previous studies (Dziobak et al., 2021; Hart et al., 2018), analyses examining demographic and environmental covariates were limited to this metabolite. A “detect” was defined as an individual with phthalate metabolite concentrations above the analytical limit of detection (LOD), and a “non‐detect” was defined as an individual with these metabolite concentrations below the LOD. Censored statistics, including Robust Regression on Order Statistics (ROS) and Kaplan‐Meier (K‐M) from the NADA package (version 1.6–1.1; Lee, 2020), were applied to calculate the mean and standard deviation (s.d.) for metabolite concentrations when sampled individuals in a group (i.e., overall, year, sex, age) had concentrations below the LOD (Dziobak et al., 2021; Helsel, 2005; Hites, 2019). Specifically, ROS was used to analyze data when less than or equal to 20% of observations were censored, whereas K‐M was used when 80% or more of observations were censored (Dziobak et al., 2021; Helsel, 2005). For dolphins with detectable metabolite concentrations (values > LOD), 95% confidence intervals (95% CI) were constructed using Wilson's (1927) method.

Kendall's tau was employed to evaluate correlations between MEHP concentrations and age, as well as between MEHP concentrations and 3‐day and 15‐day antecedent rainfall (Helsel, 2012), using the *censReg* package (version 0.5–38; Henningsen, 2024), while accounting for censored observations (Helsel, 2012). Using the *VGAM* package (Yee, 2015; Yee & Wild, 1996), bivariate Tobit regression models were used to examine MEHP differences by sex, year (decade), and laboratory, and to investigate linkages with environmental factors (i.e., antecedent rainfall, storm events, HAB events; Tobin, 1958). We grouped concentrations by decade to assess differences over time, given the long study period and large sample size. Based on significant environmental findings from bivariate Tobit regression models, multiple Tobit models were used to investigate the effect of multiple environmental factors on phthalate exposure while controlling for the sampling laboratory (Tobin, 1958) for the pooled data. Metabolite concentration data were log‐transformed, resulting in normally distributed residuals. Multicollinearity in multiple Tobit regression models was assessed using the variance inflation factor (VIF; Thompson et al., 2017) from the *car* package (Fox & Weisberg, 2019). When multicollinearity was detected between predictors (VIF >1), the predictor with the higher p‐value was excluded (Akinwande et al., 2015). A likelihood ratio test was used to assess the fit of the regression models (Neyman & Pearson, 1928), using the *VGAM* package (Yee, 2015; Yee & Wild, 1996), and statistical significance was evaluated at *α* = 0.05. Given that urine was screened by two separate labs using different extraction methods, we conducted a sensitivity analysis by running the environmental Tobit models on each lab's data to compare pooled model findings with within‐lab findings to assess robustness and ensure the pooled findings were representative by lab.

## 3 Results

### 3.1 Study Sample

This study accounted for only an individual's most recent sampling event, resulting in one sample per dolphin. Of these 138 dolphins, 73 were female and 65 were male, with ages ranging from more than one year to 49, averaging 16.5 years (Table 1). The average predicted age of sexual maturity for Sarasota Bay dolphins is 8.5 years for females and 10 years for males (Wells et al., 2025). Based on these figures, there were approximately 45 immature and 93 mature individuals, evenly distributed by sex (female = 25, 48; male = 20, 45). Health assessments were not conducted during 1996, 2007, 2020, or 2021; however, the 30‐year study covered most sampling years, representing the dolphin community over time. By lab, demographics remained representative of the overall study sample (*n* = 138; Table 1).

**Table 1:** Characteristics of Bottlenose Dolphins (*n* = 138) Analyzed for Urinary Phthalate Metabolite Concentrations, Sarasota Bay, FL, 1993–2024

Characteristic	Combined study sample		Laboratory 1 (*n* = 67)		Laboratory 2 (*n* = 71)	
	Count	Percent of total	Count	Percent of total	Count	Percent of total
Sex (F)	73	52.90	35	52.24	38	53.52
Sex (M)	65	47.10	32	47.76	33	46.48
Decade (1990s)	18	13.04	18	26.87	0	0
Decade (2000s)	48	34.78	48	71.64	0	0
Decade (2010s)	49	35.51	1	1.49	48	67.61
Decade (2020s)	23	16.67	0	0	23	32.39
Age (yrs)	Mean = 16.52	Standard deviation = 11.49	Mean = 18.41	Standard deviation = 11.43	Mean = 14.74	Standard deviation = 11.33

### 3.2 Overall Phthalate Exposure

During 1993–2024, 86.23% (*n* = 119) of Sarasota Bay dolphin urinary samples (*n* = 138) had detectable phthalate metabolite concentrations (95% CI = 79.34–91.50%). Average detectable phthalate metabolite concentrations in this study ranged from 0.17 to 6.19 ng mL^−1^, and individual concentrations ranged from <LOD to 491.00 ng mL^−1^ (Table 2). Of the eight metabolites included in this study, MBzP (*n* = 3; 2.17% detection, 95% CI = 0.74–6.20%) and MMP (*n* = 2; 1.45% detection, 95% CI = 0.40–5.13%) were detected the least, and MBP (K‐M mean = 0.17 ng mL^−1^) and MEOHP (ROS mean = 0.52 ng mL^−1^) had the lowest average concentrations (Table 2). MiBP was not detected in any samples and was excluded from further analysis. The highest average concentrations came from two metabolites of DEHP: MEHP (ROS mean = 6.19 ng mL^−1^) and MEHHP (ROS mean = 4.13 ng mL^−1^; Table 2). Overall, MEHP was the predominant metabolite, detected in 78.26% (*n* = 108; ROS mean = 6.19 ng mL^−1^; ROS s.d. = 11.29 ng mL^−1^) of samples, followed by MEP, detected in 57.97% (*n* = 80; ROS mean = 1.69 ng mL^−1^; ROS s.d. = 1.98 ng mL^−1^; Table 2) of samples. Of the two most detected metabolites, MEHP was, on average, more variable than MEP (<LOD to 76.60 ng mL^−1^ vs. <LOD to 21.20 ng mL^−1^; Table 2; Figure 2). Since MEHP was the most prevalent, these results reaffirmed limiting further analyses to this phthalate metabolite.

**Table 2:** Descriptive Statistics (*n* = 138) of All Detectable Bottlenose Dolphin Urinary Phthalate Metabolite Concentrations (ng mL^−1^; i.e., >LOD), Sarasota Bay, FL, 1993–2024

	MBP^a^	MBzP^b^	MMP^b^	MEHHP^a^	MEOHP^a^	MEHP^a^	MEP^a^	Any metabolite
Mean^c^	0.17	0.68	3.67	4.13	0.52	6.19	1.69	–
Standard deviation^a^/standard error^b^	0.19	0.11	4.71	42.25	5.96	11.29	1.98	–
# detects	33	3	2	69	34	108	80	119
% detects (95% CI)	23.91 (17.56–31.67)	2.17 (0.74–6.20)	1.45 (0.40–5.13)	50.00 (41.77–58.23)	24.64 (18.20–32.45)	78.26 (70.66–84.33)	57.97 (49.63–65.88)	86.23 (79.34–91.50)
Range^d^ (min‐max)	0.16–1.42	0.56–11.30	0.32–462.00	0.004–491.00	0.0005–70.00	0.04–76.60	0.43–21.20	–

^a^ Calculated using ROS.

^b^ Calculated using Kaplan‐Meier (K‐M).

^c^ Calculated among all samples using censoring statistics, where appropriate.

^d^ Determined only among detects.

**Figure 2:**
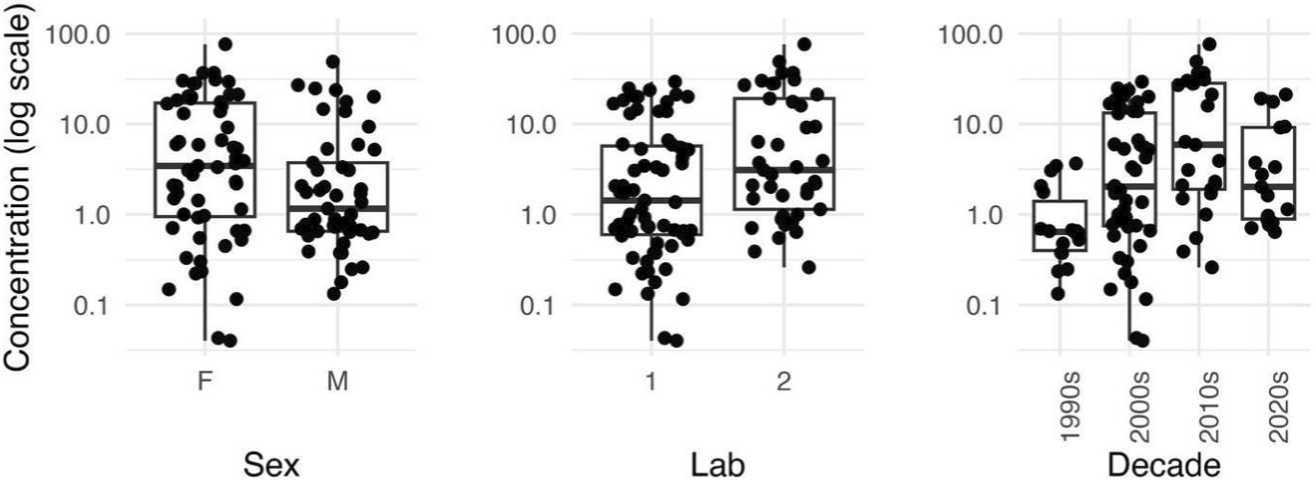
Comparisons of log‐transformed detectable MEHP concentrations (i.e., >LOD) among bottlenose dolphins from Sarasota Bay, FL (1993–2024) by sex, year (decade), and analytical laboratory. Boxplots depict medians and quartiles.

MEHP was not significantly associated with age (MEHP *τ* = −0.06; *p* = 0.29) or sex (*p* > 0.05; Table 3; Figure 2), even though the average MEHP concentration for females (ROS mean = 8.10 ng mL^−1^; ROS s.d. = 13.03) was more than twice that for males (ROS mean = 4.03 ng mL^−1^; ROS s.d. = 8.54). MEHP concentrations varied over time, showing a general increasing trend from the 1990s to the 2010s (Figure 2). Lab 1 analyzed samples from 1993 to 2010, while Lab 2 analyzed those from 2010 to 2024 (Table 3). Across laboratories, 100% of samples analyzed by Lab 1 had detectable MEHP concentrations, whereas only 57.75% of samples analyzed by Lab 2 did (Table 3), due in part to differences in lab instrument sensitivity. Bivariate Tobit regression revealed significant differences in MEHP concentrations between labs (*p* = 0.03; Lab 1: ROS mean = 6.96 ng mL^−1^, Lab 2: ROS mean = 5.37 ng mL^−1^; Table 3). When grouped by decade, the mean value for MEHP increased six‐fold from the 1990s (ROS mean = 1.13 ng mL^−1^; ROS s.d. = 1.15) to the 2000s (ROS mean 6.62 ng mL^−1^; ROS s.d. = 8.21) with a significance level of *p* = 0.05. The 2010s and 2020s decades did not differ for MEHP (*p* > 0.05; Table 3; Figure 2).

**Table 3:** Descriptive Statistics and Significance of Detectable Bottlenose Dolphin Urinary Mono(2‐Ethylhexyl) Phthalate (MEHP) Concentrations (ng mL^−1^; i.e., >LOD) by Sex, Year (Decade), and Analytical Laboratory, Sarasota Bay, FL, 1993–2024

	N	Number of detects	Percent > LOD (95% CI)	Mean^a^	Standard deviation^a^	Range^b^ (min‐max)	p^c^
Sex (F)	73	59	80.82 (70.34–88.22)	8.10	13.03	0.04–76.60	–
Sex (M)	65	49	75.38 (63.69–84.24)	4.03	8.54	0.13–49.20	0.06
Decade (1990s)	18	18	100.00 (82.41–100.00)	1.13	1.15	0.13–3.66	–
Decade (2000s)	48	48	100.00 (92.59–100.00)	6.62	8.21	0.04–29.48	0.05
Decade (2010s)	49	25	51.02 (37.47–64.42)	8.55	16.13	0.26–76.60	0.91
Decade (2020s)	23	17	73.91 (53.53–87.45)	4.22	6.55	0.64–21.30	0.41
Lab 1	67	67	100.00 (94.58–100.00)	5.37	7.63	0.04–29.48	–
Lab 2	71	41	57.75 (46.15–68.55)	6.96	13.90	0.26–76.6	**0.03**

*Note*. The bold means that these values are significant..

^a^ Calculated using ROS.

^b^ Determined only among detects.

^c^ From bivariate Tobit regression.

### 3.3 Environmental Influences on MEHP Exposure

#### 3.3.1 Rainfall Influences on MEHP Exposure

During 1993–2024, 3‐day average antecedent rainfall ranged from 0 to 1.06 inches, and 15‐day average antecedent rainfall ranged from 0 to 0.764 inches. Controlling for sampling lab, Kendall's tau showed that MEHP concentrations among dolphins were positively correlated with 3‐day average antecedent rainfall (*p* = 0.0001; *τ* = 0.20; Figure 3), but no significant differences were observed between MEHP concentrations among dolphins and 15‐day average antecedent rainfall (*p* = 0.29; *τ* = 0.06). Bivariate Tobit regression results (provided in Table S3 in Supporting Information S1) also revealed a positive association between MEHP concentrations among dolphins and 3‐day average antecedent rainfall (*p* = 0.0008), with relatively low variation (standard error [s.e.] = 0.83).

**Figure 3:**
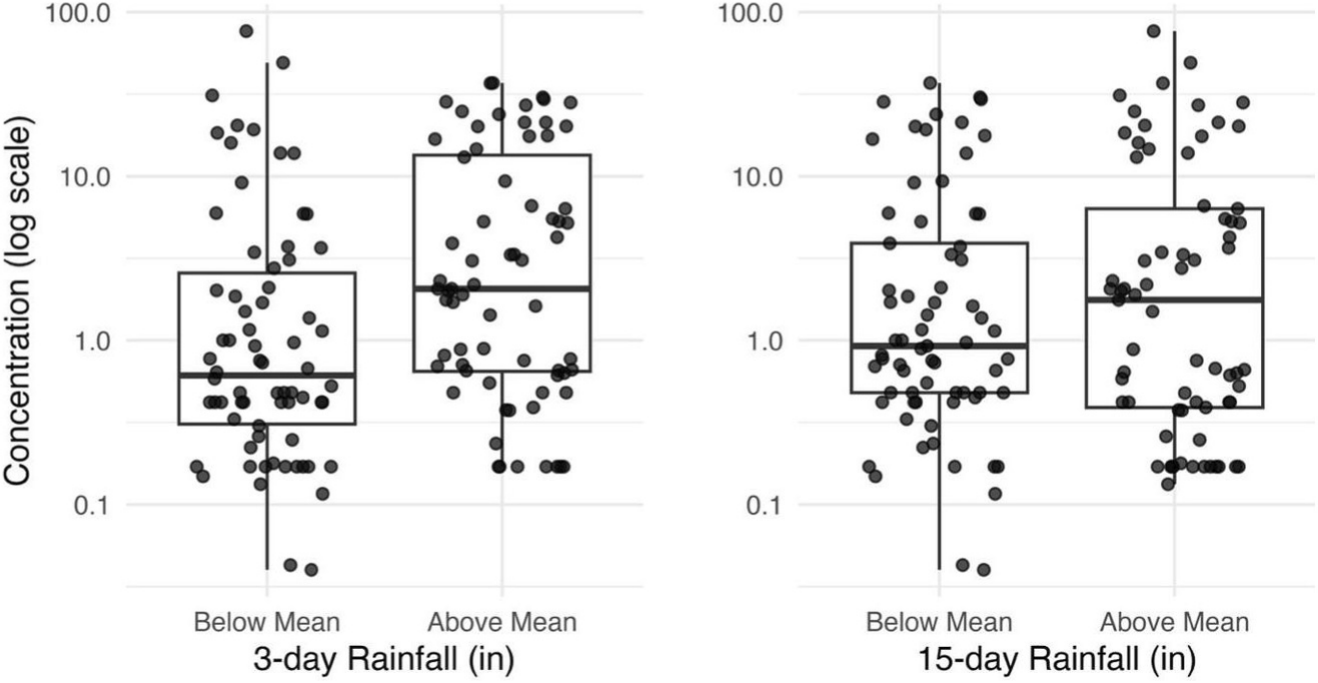
Comparisons of log‐transformed detectable MEHP concentrations (i.e., >LOD) among bottlenose dolphins from Sarasota Bay, FL (1993–2024) relative to antecedent rainfall within 3 and 15 days of sampling. The mean 3‐day average antecedent rainfall is 0.138 in, and the mean 15‐day average antecedent rainfall is 0.134 in. There were no significant differences in mean detectable MEHP concentrations between below‐mean and above‐mean rainfall values for both 3‐day and 15‐day average antecedent rainfall. Boxplots depict medians and quartiles.

#### 3.3.2 Storm Event Influences on MEHP Exposure

During the study period (1993–2024), 30 storm events occurred, and 14 individuals were sampled within six months of a storm event, 61 within nine months, and 73 within 12 months. Controlling for sampling lab, bivariate Tobit regression revealed that MEHP concentrations were significantly lower among dolphins that were sampled within six months of a storm event (*n* = 14, ROS mean = 1.00 ng mL^−1^) compared to MEHP concentrations among dolphins that were not sampled within six months of a storm event (*n* = 124, ROS mean = 6.77 ng mL^−1^; estimate = 0.16; s.e. = 0.64; *p* = 0.004; Table 4; Figure 4; Table S4 in Supporting Information S1). Other bivariate Tobit regression results are presented in Table S4 in Supporting Information S1. This finding was not observed for dolphins sampled within 9 months (*p* = 0.92) or 12 months of a storm event (*p* = 0.56; Table 4; Figure 4).

**Table 4:** MEHP Detection (Number > LOD) and Concentrations (ng mL^−1^) Among Bottlenose Dolphins Sampled Within Six, Nine, and 12 Months (Yes) of a Storm Event Versus Outside of Six, Nine, and 12 Months (No) of a Storm Event, Sarasota Bay, FL, 1993–2024

	Storm event 6 months		p^a^	Storm event 9 months		p^a^	Storm event 12 months		p^a^
	Yes (*n* = 14)	No (*n* = 124)		Yes (*n* = 61)	No (*n* = 77)		Yes (*n* = 73)	No (*n* = 65)	
**# >LOD**, %, (95% CI)	**8**, 57.14 (32.59–78.62)	**100**, 80.65 (72.81–86.63)	–	**50**, 81.97 (70.53–89.62)	**58**, 75.32 (64.65–83.60)	–	**61**, 83.56 (73.43–90.34)	**47**, 72.31 (60.42–81.71)	–
Mean (standard deviation)^b^	1.00 (1.34)	6.77 (11.76)	**0.004**	4.54 (7.10)	7.49 (13.64)	0.92	6.61 (12.56)	5.71 (9.75)	0.56

*Note*. The bold means that these values are significant..

^a^ p‐values represent bivariate Tobit results analyzed independently by storm event period.

^b^ Calculated using ROS.

**Figure 4:**
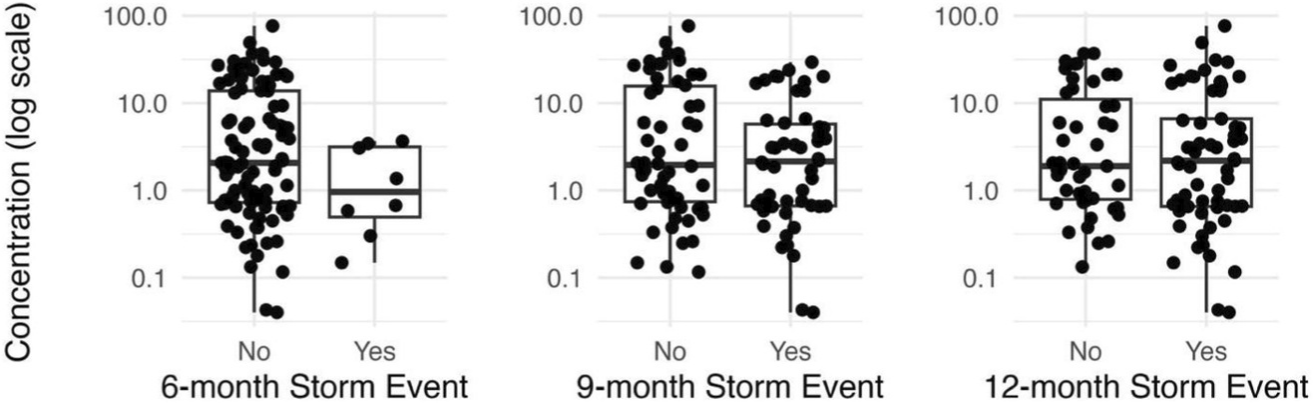
Comparisons of log‐transformed detectable MEHP concentrations (i.e., >LOD) among bottlenose dolphins from Sarasota Bay, FL (1993–2024) sampled within six, nine, and 12 months of a storm event (Yes) versus outside of six, nine, and 12 months of a storm event (No). Boxplots depict medians and quartiles.

#### 3.3.3 Harmful Algal Bloom Influences on MEHP Exposure

During the study period, there were a total of 15 HAB events that exceeded fish kill levels. Forty‐six individuals were sampled within three months of a HAB event, 63 individuals within six months, 66 individuals within nine months, and 81 individuals within 12 months. There were no statistically significant differences in average MEHP concentrations among dolphins sampled within three, six, nine, or 12 months of a HAB event (ROS mean: 3.93–4.84 ng mL^−1^) compared to MEHP concentrations among dolphins that were not sampled within these timeframes of a HAB event (ROS mean: 6.88–9.40 ng mL^−1^; Table 5; *p* > 0.05; Figure 5) when controlling for lab. Other bivariate Tobit regression results are presented in Table S5 in Supporting Information S1.

**Table 5:** MEHP Detection (Number > LOD) and Concentrations (ng mL^−1^) Among Bottlenose Dolphins Sampled Within Three, Six, Nine, and 12 Months (Yes) of a Storm Event Versus Outside of Six, Nine, and 12 Months (No) of a HAB Event, Sarasota Bay, FL, 1993–2024

	HAB event 3 months		p^a^	HAB event 6 months		p^a^	HAB event 9 months		p^a^	HAB event 12 months		p^a^
	Yes (*n* = 46)	No (*n* = 92)		Yes (*n* = 63)	No (*n* = 75)		Yes (*n* = 66)	No (*n* = 72)		Yes (*n* = 81)	No (*n* = 57)	
**# >LOD**, %, (95% CI)	**29**, 63.04 (48.60–75.48)	**79**, 85.87 (77.31–91.55)	–	**45**, 71.43 (59.30–81.10)	**63**, 84.00 (74.08–90.60)	–	**47**, 71.21 (59.36–80.73)	**61**, 84.72 (74.68–91.25)	–	**58**, 71.60 (60.98–80.27)	**50**, 87.72 (76.75–93.92)	–
Mean (standard deviation)^b^	4.84 (7.45)	6.88 (12.76)	0.35	4.08 (6.55)	7.97 (13.89)	0.45	3.95 (6.43)	8.25 (14.10)	0.37	3.93 (6.45)	9.40 (15.30)	0.29

*Note*. The bold means that these values are significant.

^a^ p‐values represent bivariate Tobit results analyzed independently by HAB event period.

^b^ Calculated using ROS.

**Figure 5:**
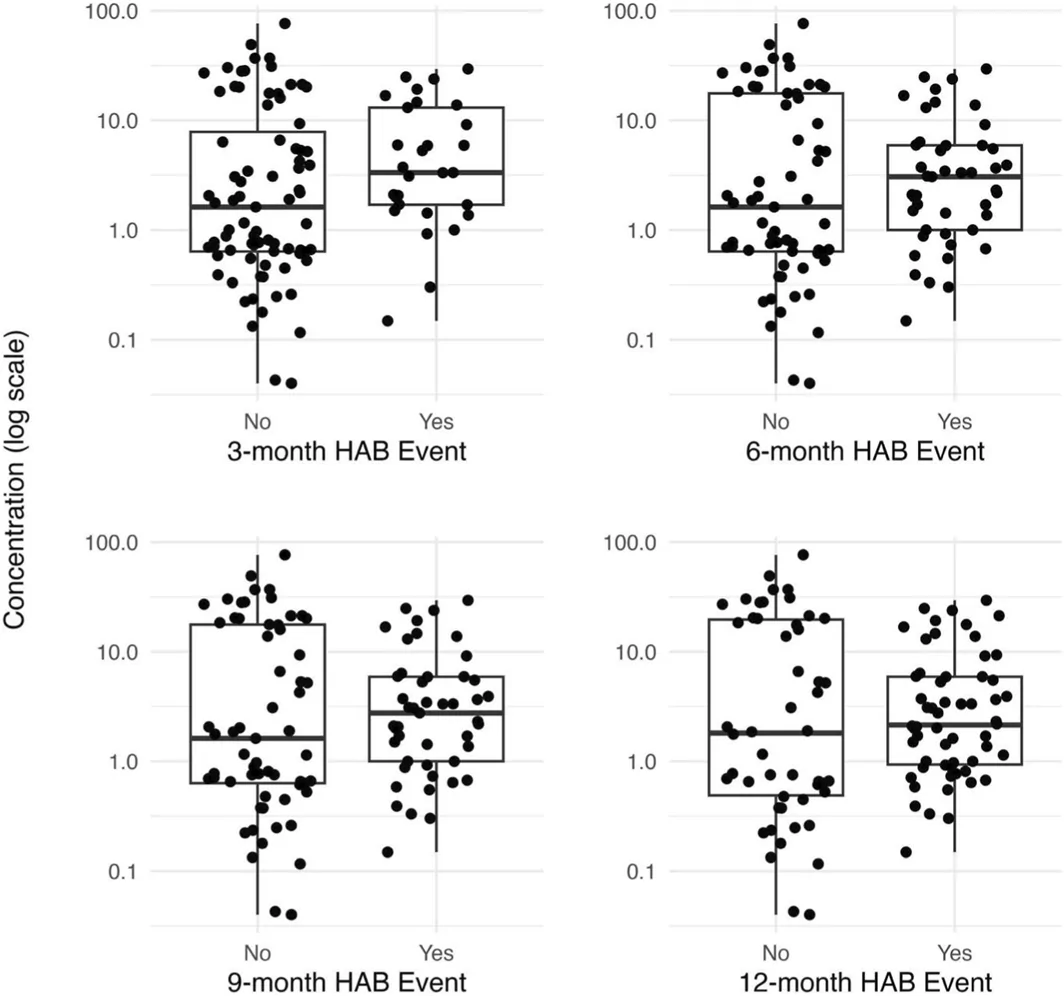
Comparisons of log‐transformed detectable MEHP concentrations (i.e., >LOD) among bottlenose dolphins from Sarasota Bay, FL (1993–2024) sampled within three, six, nine, and 12 months of a harmful algal bloom (HAB) event (Yes) versus outside of three, six, nine, and 12 months of a HAB event (No). Boxplots depict medians and quartiles.

#### 3.3.4 Combined Influences on MEHP Exposure

Three‐day average antecedent rainfall and 6‐month storm events yielded statistically significant results from bivariate Tobit regression models and were, therefore, included to evaluate the combined influences of these environmental factors on MEHP exposure in dolphins. While controlling for differences in concentrations between analytical labs (*p* = 0.03; Table 3), MEHP concentrations remained significantly higher among dolphins that were sampled following 3‐day average antecedent rainfall (*p* = 0.01) and significantly lower among dolphins that were sampled within 6 months of a storm event (*p* = 0.007; Table 6). No collinearity was detected in the multiple Tobit model (Akinwande et al., 2015), and a likelihood ratio test indicated that this model provided a significantly better fit than the null (*p* = 0.0002; Table 6; Neyman & Pearson, 1928). Sensitivity analysis reaffirmed the directional relationship of the pooled model results, with MEHP concentrations significantly higher following 3‐day average antecedent rainfall and lower within 6 months of a storm event across both labs, though not statistically significant for Lab 1 (Table 7).

**Table 6:** Multiple Tobit Regression Results From Combined Environmental Influences on Log‐Transformed MEHP Concentrations Among Bottlenose Dolphins While Accounting for Sampling Laboratory, Sarasota Bay, FL, 1993‐2024

Variable	Estimate^a^	Standard error	p
Storm event 6 months	0.18	0.63	**0.007**
Rainfall 3‐day	8.25	0.84	**0.01**
Lab	0.52	0.36	0.07

*Note.* Log‐likelihood = −248.78; model versus null *p* = 0.0002. The bold means that these values are significant.

^a^ Back‐transformed estimate.

**Table 7:** Sensitivity Analysis by Lab for Multiple Tobit Regression Results From Combined Environmental Influences on Log‐Transformed MEHP Concentrations Among Bottlenose Dolphins, Sarasota Bay, FL, 1993‐2024

	Variable	Estimate^a^	Standard error	p
Laboratory 1	Storm event 6 months	0.74	0.61	0.62
	Rainfall 3‐day	6.02	1.79	**0.02**
Laboratory 2	Storm event 6 months	0.11	0.78	**0.005**
	Rainfall 3‐day	12.53	1.28	**0.05**

*Note*. The bold means that these values are significant.

^a^ Back‐transformed estimate.

## 4 Discussion

### 4.1 Overall Phthalate Exposure

The objective of this study was to assess temporal trends in phthalate exposure across the Sarasota Bay common bottlenose dolphin community and determine whether exposure was associated with environmental factors, including antecedent rainfall, storm events (i.e., tropical storms, tropical depressions, hurricanes), and HABs. Evidence of phthalate exposure in more than 86% of dolphins sampled substantiates pervasive contamination in this estuarine system. Detection variability across phthalate metabolites was expected, given that sampling was independent of the timing of exposure, and phthalates vary in structure, molecular weight, application, and environmental fate (Staples et al., 1997) and are, in turn, metabolized and excreted from organisms at different rates (Y. Zhang et al., 2021). It was not surprising that MEHP was the dominant metabolite detected in this study sample, observed in more than 78% of the urine samples, considering the historically high production volume (Qu et al., 2022), widespread coastal and marine environmental contamination (Paluselli & Kim, 2020; Paluselli et al., 2018; L. Y. Wang et al., 2021; Z. M. Zhang et al., 2020), and slow degradation (Henkel et al., 2023) of its parent compound, DEHP. MEHP showed no associations with sex or age (*p* > 0.05), supporting past findings despite the increased sample size and study duration of the present study, suggesting no demographic predisposition to exposure across all Sarasota Bay bottlenose dolphins (Dziobak, Wells, et al., 2022; Dziobak et al., 2021; Hart et al., 2020). The lack of association with age could also imply continuous phthalate exposure, but limited bioaccumulation of the contaminant (Staples et al., 1997). Additionally, significant differences in concentrations between the two labs could reflect environmental contamination differences associated with increased plastic production over time or temporal changes in sampling protocols, given that all samples were analyzed in 2024. Additionally, any effects from these confounding variables were minimized by applying censoring statistics to account for values below the LOD in Lab 2 (Dziobak et al., 2021; Helsel, 2005; Hites, 2019).

Evidence of long‐term MEHP exposure (1993–2024) in the Sarasota Bay bottlenose dolphin community indicates persistent, continuous phthalate contamination in estuarine environments, suggesting chronic ecotoxicological effects (Kucklick et al., 2011; Wells et al., 2004). Urinary MEHP levels rose significantly from the 1990s to the 2000s decade and showed an increasing, but non‐significant, temporal trend from the 1990s through the 2010s, aligning with the rise in global phthalate production over time (Godwin, 2017). Aside from the 2000s decade, the non‐significant differences in MEHP concentrations over time contradict the temporal findings reported by Dziobak, Balmer, et al. (2022), who found significantly higher urinary MEHP levels in 2014–2015 than in other years between 2010 and 2019. It is likely that the longer time period and larger sample size in this study diluted the temporal differences observed in Dziobak, Balmer, et al. (2022), given that the samples were grouped by decade. These results highlight the persistence of MEHP and DEHP, specifically, in estuarine environments. Sustained MEHP concentrations in this study sample over 30 years may also indicate that production bans and regulations primarily targeting DEHP, initiated in 2008 in the U.S. (U.S. Consumer Product Safety Improvement Act [CPSIA], 2008), have not sufficiently reduced phthalate contamination in the environment, potentially underscoring the need for stricter regulations on production, imports, and disposal to prevent further environmental phthalate contamination. A slight, but non‐significant decline in MEHP concentrations from the 2010s to 2020s decade could also reflect the lag time in production bans that have led to increasing industry replacements of DEHP with other phthalate mixtures (DIDP and DINP; European Chemicals Agency [ECHA], 2013; Kim et al., 2021; Wicke et al., 2021; Zota et al., 2014), but further monitoring is needed.

### 4.2 Environmental Influences on MEHP Exposure

Historically, NOAA's Atlantic hurricane season began in June, and the dry season ended in May (NOAA, n.d.). While timing differences could have impacted the number of storm and HAB events that occurred within three, six, or nine months each sample date, as well as the amount of 3‐day and 15‐day average antecedent rainfall, it should be noted that, in this study sample, 63 individuals were sampled during the wet season (June–October), while 75 individuals were sampled during the dry season (November–May). The relatively even distribution between the two seasons highlights that this large sample size (*n* = 138) reflects long‐term environmental trends in Sarasota Bay.

#### 4.2.1 Rainfall Influences on MEHP Exposure

A positive association between urinary MEHP concentrations in Sarasota Bay bottlenose dolphins and average rainfall occurring 3 days before sampling indicates that short‐term rainfall may trigger acute phthalate exposure events in Sarasota Bay and other coastal urbanized estuaries. These findings are similar to those of Müller et al. (2019), in which one‐day rainfall events elevated phthalate levels in runoff. Increased MEHP levels following 3‐day average antecedent rainfall is also supported by past studies showing that estuarine phthalate inputs were significantly higher in the rainy season than in the dry season due to atmospheric deposition (Gao & Wen, 2016), contaminated runoff (Weizhen et al., 2020) from landfills (Gholaminejad et al., 2024; Mohammadi et al., 2022), and urban, industrial, and residential land use (Cao et al., 2022; Wicke et al., 2021). Additionally, the lack of correlation between MEHP concentrations and 15‐day average antecedent rainfall is likely due to initial runoff peaks followed by exponential decay over time after a rainfall event (Egodawatta, 2007). Additionally, the overall maximum amount of 3‐day average antecedent rainfall was greater than that for 15‐day average antecedent rainfall, supporting the notion of an initial washout of phthalates through runoff. Overall, phthalate exposure in this sentinel species may reflect short‐term fluctuations in environmental contamination (Kucklick et al., 2011; Wells et al., 2004). Further research should be conducted to identify land‐based sources that contribute to contaminated runoff and increased estuarine phthalate exposure during short‐term precipitation events.

#### 4.2.2 Storm Event Influences on MEHP Exposure

Although the intensity of storm events (e.g., hurricane category) was not considered in this analysis, significantly lower urinary MEHP concentrations were still observed in Sarasota Bay dolphins that were sampled within 6 months of a storm event (i.e., tropical storm, tropical depression, hurricane), compared to those not sampled within any storm events during this time. These results demonstrate that large‐scale storm events may influence dolphin phthalate exposure. While past studies have found elevated phthalate levels in highly urbanized stormwater drains and catchments following storms (Cao et al., 2022; Wicke et al., 2021), aquatic phthalate concentrations have been shown to decline from inland rivers to tidally influenced estuaries (Weizhen et al., 2020). It is possible that both the increased distance from these marine phthalate sources and the dilution effect observed during the rainy season (C. F. Chen et al., 2022; Heo et al., 2020) contributed to lower MEHP levels in dolphins 6 months after large‐scale storm events. These findings are also supported by past studies reporting reduced DEHP exposure in humans 45 days to up to 1 year (Watkins et al., 2024) and 6 months (Samon et al., 2022) following a hurricane.

However, considering the positive association with 3‐day average antecedent rainfall, other characteristics of large‐scale storms, such as turbulence, could potentially contribute to altered coastal phthalate contamination. For example, large‐scale storm events can intensify turbulence, wave action, and tidal mixing (Veeramony et al., 2020), which may resuspend phthalates from sediments and transport them from the coast into the open ocean (Billings et al., 2024; Mi et al., 2023), resulting in lower concentrations in coastal estuaries. The lack of associations between MEHP concentrations and storm events occurring within nine or 12 months before sampling may suggest that tropical weather patterns preceding sampling by more than 6 months do not affect phthalate exposure in dolphins and may support the notion that phthalates are unlikely to bioaccumulate in their tissues (Staples et al., 1997), as they are ultimately excreted in urine. While DEHP, in particular, can be stored in organs, it is rapidly metabolized and excreted via urine in humans and rodents (Koch et al., 2005; Silva et al., 2006). Therefore, exposure in Sarasota Bay bottlenose dolphins, an apex keystone species, could result from continuous ingestion of contaminated prey or inhalation (Conger et al., 2024; Dziobak et al., 2024; Hart et al., 2022, 2023). Further studies should consider the impacts of large‐scale storms on offshore marine phthalate exposure, particularly DEHP and its primary metabolite MEHP, as well as storm intensity and associated characteristics on estuarine phthalate exposure and contamination risks.

#### 4.2.3 Harmful Algal Bloom Influences on MEHP Exposure

The lack of association between MEHP concentrations among bottlenose dolphins and HAB events suggests that HABs may not influence phthalate exposure or contamination and may be explained by two different ecological mechanisms. First, phthalates can prevent marine algal growth (Savoca et al., 2026; Shen et al., 2019; Wenchao et al., 2023). *K. brevis*, one of the algal organisms responsible for HABs in the Gulf of Mexico, is no exception, as phthalates can inhibit its growth by more than 80% (N. Liu et al., 2016). However, phthalates are primarily removed from the environment through biodegradation (Paluselli et al., 2019; Staples et al., 1997; Suhrhoff & Scholz‐Böttcher, 2016), a process facilitated by marine algae. For instance, *K. brevis* can rapidly degrade phthalates (Sun et al., 2019), but metabolite byproducts created by these primary producers during the degradation process may enhance bioavailability risk to consumers (Savoca et al., 2026). These studies illustrate the contrasting relationship between *K. brevis* and phthalates, and the non‐significant findings from this study may reflect this complexity. The results from this study also imply that estuarine phthalate contamination during a *K. brevis* bloom may be similar to non‐bloom conditions.

Secondly, temporary shifts in dolphin prey distribution (Berens McCabe et al., 2021; Gannon et al., 2009; Wells et al., 2013) and in foraging behavior (McHugh et al., 2011) have been well documented during HAB events. We suspected these changes could influence phthalate exposure, since recent studies have demonstrated plastic contamination among their common prey species (Conger et al., 2024; Hart et al., 2022, 2023). Based on this study's findings, HAB events do not appear to influence MEHP exposure in Sarasota Bay bottlenose dolphins. These findings reaffirm the ubiquity of phthalates in the estuarine and marine environment, potentially indicating pervasive exposure across the food web. Further studies should examine phthalate exposure in abundant and common dolphin prey, such as pinfish (*Lagodon rhomboides*) and pigfish (*Orthopristis chrysoptera*), relative to clupeids (*Clupeidae* spp.), which are the dominant prey during HAB conditions (Berens McCabe et al., 2021; Gannon et al., 2009; McHugh et al., 2011).

#### 4.2.4 Combined Environmental Influences on MEHP Exposure

The resulting combined associations among MEHP concentrations in Sarasota Bay dolphins, 3‐day average antecedent rainfall (*p* = 0.01), and 6‐month storm events (*p* = 0.007) indicate that short‐term rainfall and large‐scale storms differentially impact MEHP exposure in dolphins and, consequently, contamination in the Sarasota Bay estuary. The within‐lab sensitivity analysis reaffirmed these findings and validated the use of the pooled data set over the 30‐year period. However, the lab differences for 6‐month storm events could be due to smaller sample sizes than in the pooled data set, resulting in lower power, or to the lack of censoring required in Lab 1, which would lead to less variability. Despite this, the direction of the relationships was maintained within each lab, as determined by the estimates and p‐values, suggesting that lab differences did not confound the analysis. Short‐term rainfall may have a greater effect on phthalate exposure and contamination in Sarasota Bay, given the temporal proximity to sampling, but the negative association with storm events should be explored further. Additionally, these findings indicate that weather‐induced chemical mobilization of phthalates may be reflected in dolphin exposure. Findings from this study could also imply similar exposure risks for other dolphin communities inhabiting storm‐prone, urban, subtropical estuaries. Furthermore, these results suggest that other organic pollutants with physicochemical properties similar to those of phthalates (e.g., bisphenol A [BPA], polychlorinated biphenyls [PCBs], organochlorine or organophosphate pesticides) may behave similarly in response to these environmental factors.

Overall, this study demonstrates that MEHP exposure in a marine sentinel species is widespread within a population, persistent, and influenced by environmental conditions that are expected to worsen over time. For example, climate projections indicate a significant rise in tropical cyclone risks for the U.S. Gulf Coast, amplifying storm surge and sea level rise in Sarasota Bay (Frazier et al., 2010; Marsooli et al., 2019). While more frequent large‐scale storm events may lead to reduced MEHP exposure and contamination along the Sarasota Bay coast, more intense short‐term precipitation events associated with these weather patterns could also exacerbate estuarine MEHP exposure and contamination, especially if the production and use of these endocrine‐disrupting parent compounds persist. Thus, these environmental findings could also provide support for policy changes and legal protections for bottlenose dolphins and their habitats. Dolphin habitats, including mangroves and seagrass meadows, are particularly vulnerable to sea level rise and intense storm events, and increased pollutant loads in estuarine waters could have compounding effects (Tomasko et al., 2020). Although toxicity remains uncertain for exposed bottlenose dolphins, there are indications of hormone disruption in this community (Dziobak, Wells, et al., 2022), and numerous adverse effects have been observed in sensitive aquatic and marine biota (Y. Liu et al., 2023; Mankidy et al., 2013; Molino et al., 2019; Seyoum & Pradhan, 2019; S. Wang et al., 2020; Ye et al., 2014). Therefore, this study highlights the importance of conserving critical estuarine habitats from known endocrine disruptors and the need for further research on the health impacts to bottlenose dolphins and other marine mammals.

## 5 Conclusion

To our knowledge, this is the most comprehensive study on phthalate exposure in marine mammals to date. The long‐term monitoring of bottlenose dolphins residing in Sarasota Bay, FL, as well as critical long‐term public environmental monitoring data, enabled our investigation into some environmental factors influencing phthalate exposure in this sentinel species. The reliance on archived urine samples and previously analyzed data, combined with an extensive sample size spanning 30 years, enabled robust and efficient analyses that revealed widespread evidence of phthalate exposure. Past work documenting exposure in Sarasota Bay bottlenose dolphins also provided an opportunity to build upon these findings and to apply and refine methods relevant to this study. This study reaffirms long‐term MEHP exposure in Sarasota Bay bottlenose dolphins, implying persistent and continuing phthalate contamination in this estuarine environment. Distinct associations with average antecedent rainfall and large‐scale storm events (i.e., tropical storms, tropical depressions, hurricanes) indicate that environmental factors influence MEHP exposure in Sarasota Bay bottlenose dolphins and may potentially impact phthalate exposure in other marine or estuarine species. While the full health impacts on dolphins are not yet understood, long‐term phthalate exposure in this apex keystone species and its connections to environmental variables are critical for future conservation work. Worsening environmental conditions may exacerbate phthalate exposure and contamination in urban, subtropical estuaries that experience frequent, heavy rainfall, such as Sarasota Bay. Therefore, it might be prudent to take appropriate steps to mitigate exposure to and contamination by such organic pollutants with endocrine‐disrupting properties.

## Conflict of Interest

The authors declare no conflicts of interest relevant to this study.

## Supporting information

Supporting Information S1

## Data Availability

All data used for this study's analysis are available through the Open Science Framework via https://doi.org/10.17605/OSF.IO/T6X2P. All raw environmental data used to support this study's analysis are publicly available. Refer to Section 2.3 Environmental Data Collection for more information on obtaining environmental data. Geographic sighting data were also used to generate Figure 1.
