# Supplementary material for: Exploring Environmental Influences on Mono(2‐Ethylhexyl) Phthalate (MEHP) Exposure in Common Bottlenose Dolphins (Tursiops truncatus) in Sarasota Bay, FL, USA
Source: Geohealth. 2026 Nov 6;10(11):e2026GH002199. doi: 10.1029/2026GH002199 (PMC13743994; doi:10.1029/2026GH002199)
Supplement: Supplementary file 1 [file GH2-10-e2026GH002199-s001.pdf]

GeoHealth

Supporting Information for

Exploring Environmental Influences on Mono(2-ethylhexyl) Phthalate (MEHP) Exposure in Common Bottlenose Dolphins (*Tursiops truncatus*) in Sarasota Bay, FL, USA

K. B. Warden[1], M. K. Dziobak[2,3], K. A. Wilkinson[4], R. S. Wells[4], P. A. Sandifer[5], V. M. Vulava[6], L. B. Hart[2,5]

[1]Environmental and Sustainability Studies Graduate Program, College of Charleston, Charleston, SC, USA

[2]Department of Health and Human Performance, School of Health Sciences, College of Charleston, Charleston, SC, USA

[3]Department of Environmental Health Sciences, Arnold School of Public Health, University of South Carolina, Columbia, SC, USA

[4]Brookfield Zoo Chicago's Sarasota Dolphin Research Program, Sarasota, FL, USA

[5]Center for Coastal Environmental and Human Health, College of Charleston, Charleston, SC, USA

[6]Department of Geology and Environmental Geosciences, School of Natural and Environmental Sciences, College of Charleston, Charleston, SC, USA

Contents of this file

Tables S1 to S5

Introduction

The data in these tables support the primary analysis in this paper. Tables S1 and S2 present the limits of detection for the two analytical laboratories that screened dolphin urine samples for phthalate metabolites, as reported by the laboratories. Tables S3-S5 include simple Tobit regression results, as output in R, for the three environmental factors (antecedent rainfall, large-scale storm events, and harmful algal bloom [HAB] events) that were analyzed relative to mono(2-ethylhexyl) phthalate (MEHP) concentrations.

Table S1. Limits of detection (LOD; ng mL^{-1}) for all phthalate metabolites from sampling laboratory 1.

Sample Date	MBP	MBzP	MMP	MEHHP	MiBP	MEOHP	MEHP	MEP
1993-2010	0.011112	0.002563	0.054048	0.002943	0.002222	0.002923	0.002783	0.058254

Table S2. Limits of detection (LOD; ng mL-1) for all phthalate metabolites from sampling laboratory 2.

Year	MBP	MBzP	MMP	MEHHP	MiBP	MEOHP	MEHP	MEP
2014	0.6	0.56	0.12	0.64	0.69	0.49	0.28	3.43
2010-2015; 2018-2019	0.36	0.34	0.07	0.38	0.42	0.3	0.17	1.46
2015	0.85	0.8	0.08	0.9	0.98	0.7	0.4	2.06
2016-2017	0.35	0.07	0.07	0.14	0.35	0.07	0.42	1.71
2022-2024	1.03	1.2	0.52	1.35	1.39	1.05	0.48	4.12

Table S3. Simple Tobit regression results from log-transformed MEHP concentrations among bottlenose dolphins relative to average antecedent rainfall within 3 days and 15 days of sampling while accounting for sampling laboratory, Sarasota Bay, FL, 1993-2024.

	Estimate[a]	Standard error	p
Rainfall 3-day	15.98	0.83	**0.0008**

Rainfall 15-day	4.75	1.44	0.28

[a]Back-transformed estimate.

Table S4. Simple Tobit regression results from log-transformed MEHP concentrations in the presence (Yes) vs. absence (No) of storm events within 6, 9, and 12 months of sampling while accounting for sampling laboratory, Sarasota Bay, FL, 1993-2024.

	**Estimate**[a]	**Standard error**
Storm event 6 months	0.16	0.64
Storm event 9 months	0.96	0.38
Storm event 12 months	1.25	0.38

[a]Back-transformed estimate.

Table S5. Simple Tobit regression results from log-transformed MEHP concentrations in the presence (Yes) vs. absence (No) of HAB events within 3, 6, 9, and 12 months of sampling while accounting for sampling laboratory, Sarasota Bay, FL, 1993-2024

	**Estimate**[a]	**Standard error**
HAB event 3 months	0.69	0.40
HAB event 6 months	0.75	0.38
HAB event 9 months	0.71	0.38
HAB event 12 months	0.67	0.38

[a]Back-transformed estimate.
